# Neurocognitive and Neuropsychiatric Trajectories in a Post-COVID Cohort: A Descriptive Longitudinal Study

**DOI:** 10.3390/neurolint18090163

**Published:** 2026-08-24

**Authors:** Giulia Del Duca, Marta Camici, Isabella Sperduti, Anna Clelia Brita, Martina Maresca, Carmela Pinnetti, Ilaria Mastrorosa, Valentina Mazzotta, Andrea Antinori

**Affiliations:** 1 Viral Immunodeficiency Unit, Clinical Department of Infectious Diseases and Research, National Institute for Infection Disease Lazzaro Spallanzani IRCCS, Via Portuense 292, 00149 Rome, Italy; marta.camici@inmi.it (M.C.); annaclelia.brita@inmi.it (A.C.B.); martina.maresca@inmi.it (M.M.); carmela.pinnetti@inmi.it (C.P.); ilaria.mastrorosa@inmi.it (I.M.); valentina.mazzotta@inmi.it (V.M.); andrea.antinori@inmi.it (A.A.); 2 Regina Elena National Cancer Institute IRCCS, 00144 Rome, Italy; isperduti@yahoo.it

**Keywords:** post-acute COVID-19 syndrome (PACS), long COVID, post COVID-19 condition, cognitive outcomes, neuropsychiatric symptoms, longitudinal neuropsychological evaluation

## Abstract

Introduction: Cognitive dysfunction (“brain fog”) is a common manifestation of post-acute COVID-19 syndrome (PACS) and may persist long after the acute infection. While cross-sectional studies have described cognitive deficits, longitudinal evidence on recovery trajectories remains limited. Methods: We conducted a longitudinal observational study of neurocognitive performance and neuropsychiatric symptoms in patients with PACS. Participants underwent assessment with 20 standardized tests covering five cognitive domains (memory, attention, language, executive functions, psychomotor processing speed); anxiety, depression, and sleep quality were assessed at three time points. Changes were analysed using the Friedman test. Results: Forty-two patients were included (median age 57 years; 35.7% female) from a predominantly hospitalized cohort (81% hospitalised; 66.7% requiring respiratory support). Patients who completed all three assessments (completers, n = 42) were compared with those who attended the first evaluation but did not complete follow-up (non-completers, n = 544); completers were more severely ill during the acute phase rather than healthier or more motivated. At the group level, statistically significant improvements over time were observed across the whole sample in verbal short-term learning, visuospatial memory, working memory, constructional praxis, phonological verbal fluency, and psychomotor processing speed (all *p* ≤ 0.05); after Benjamini–Hochberg adjustment across the twenty cognitive outcomes, visuospatial span forward and backward and psychomotor processing speed remained significant (all FDR-adjusted *p* ≤ 0.013), with the change confined to the first six months. Sleep quality also improved (*p* < 0.0001). Conclusion: In this cohort, group-level performance improved in six of the twenty tests administered, of which three remained significant after correction for multiple comparisons, while 28 of 42 patients (66.7%) still scored in the impaired range on at least one test at 12 months, and 17 (40.5%) on two or more. These findings highlight the importance of long-term neuropsychological monitoring and integrated cognitive-psychiatric evaluation in post-COVID care. Given the small, predominantly hospitalized sample, improvements should be interpreted cautiously and confirmed in larger controlled studies, although the use of alternate forms for part of the battery makes task-specific learning an incomplete explanation.

## 1. Introduction

Post-acute COVID-19 syndrome (PACS)—also referred to as long COVID (LC) and, in the World Health Organization (WHO) case definition, as post COVID-19 condition (PCC)—is a multisystemic condition characterized by the persistence of symptoms following the acute phase of SARS-CoV-2 infection, with more than two hundred symptoms described across multiple organ systems [1]. These symptoms may affect multiple organ systems and frequently include fatigue, cognitive difficulties, mood disturbances and sleep disorders, representing an increasingly recognized challenge for healthcare systems worldwide [2,3]. Although early case definitions (e.g., NICE 2021) considered symptoms persisting beyond four weeks from the acute infection, more recent definitions are more restrictive: those endorsed by the WHO (as post COVID-19 condition), the National Academies of Sciences, Engineering, and Medicine (NASEM) and the Centers for Disease Control and Prevention (CDC) require symptom persistence for at least three months, a criterion that improves specificity and reduces the inclusion of patients with self-limiting subacute symptoms [3,4,5].

Among the neurological manifestations of PACS, cognitive dysfunction—often described by patients as “brain fog”—is one of the most commonly reported symptoms and may substantially impair daily functioning and quality of life [6]. Cognitive symptoms may already be present during the acute illness itself. They can also emerge in the subacute phase or shortly after hospital discharge, and persist for several months following the acute phase of COVID-19 [7,8,9,10]. Large international studies have confirmed both the scale and the persistence of these deficits: a systematic review and meta-analysis estimated measurable cognitive impairment in approximately one in five individuals twelve or more weeks after infection [10], a further meta-analysis documented mid- and long-term neurological and neuropsychiatric manifestations across cohorts [11], and large community-based studies have shown measurable cognitive deficits and persistent somatic symptoms at population scale [12].

The pathophysiological mechanisms underlying cognitive impairment in patients following COVID-19 are likely multifactorial. Proposed mechanisms include neuroinflammation, immune dysregulation, endothelial dysfunction and microvascular injury, as well as indirect effects of systemic illness such as hypoxia or widespread cytokine release [13,14,15,16,17]. Previous studies have identified impairments across multiple cognitive domains, particularly executive functions, attention, memory and processing speed [18].

Although cross-sectional investigations have consistently documented cognitive deficits in individuals following COVID-19, longitudinal evidence describing the trajectory of cognitive recovery remains limited and heterogeneous. Some studies have shown that a substantial proportion of patients continue to exhibit pathological performance in at least one cognitive domain up to one year after infection [19,20]. Moreover, persistent cognitive slowing and mental fatigue have been reported even in individuals who experienced mild forms of the disease [21].

Cognitive impairment in PACS is also frequently associated with neuropsychiatric symptoms, including anxiety, depression and sleep disturbances, which may further contribute to reduced quality of life and functional limitations [22,23]. Understanding the interaction between neurocognitive deficits and psychiatric symptoms may therefore be crucial for improving long-term management strategies in these patients.

The aim of this study was to investigate longitudinal changes in neurocognitive performance and neuropsychiatric symptoms in a cohort of patients with PACS undergoing comprehensive neuropsychological assessment during follow-up.

## 2. Materials and Methods

### 2.1. Study Design and Setting

The Neuro-COVID Study is an observational, longitudinal, monocentric study conducted at the Post-COVID outpatient service of the National Institute for Infectious Diseases Lazzaro Spallanzani (IRCCS), Rome, Italy.

The study was approved by the Ethics Committee of INMI Lazzaro Spallanzani (approval number 119/2020, dated 20 May 2020) and conducted in accordance with the Declaration of Helsinki. All participants provided written informed consent prior to participation.

### 2.2. Participants

Patients with a documented history of SARS-CoV-2 infection and persistent neurological or cognitive symptoms were eligible for inclusion. Participants were either referred by clinicians or self-referred to the Post-COVID outpatient service for symptoms occurring at least four weeks after the acute infection, consistent with the definition of post-acute COVID-19 syndrome. Recruitment began in September 2020, when the operational definition applied at our service—and in the study protocol approved in May 2020—followed the early criterion of symptoms persisting at least four weeks after the acute infection, subsequently formalised in the NICE guideline of 2021. The three-month criterion later endorsed by the WHO, NASEM and CDC was published after recruitment had begun and was not applied retrospectively; inclusion criteria were not modified during the study. Throughout this article we use PACS for consistency with the wider neuropsychological literature, while recognising that the WHO, NASEM and CDC refer to the same clinical picture as post COVID-19 condition or long COVID; the divergence from those definitions concerns the symptom duration required for inclusion, not the construct itself, and the inclusion window used here reflects the case definition in force at the time of enrolment. The implications of this broader window are addressed in the Discussion.

Patients who underwent neurocognitive assessment within seven months after resolution of the acute infection were considered eligible for the present analysis. The study period extended from September 2020 to January 2023. Because the baseline (t0) assessment could be performed at any time up to seven months after the resolution of the acute infection rather than at a fixed interval, the timing of the first evaluation varied across participants. This variability in the baseline window represents a potential source of heterogeneity, as patients assessed earlier and those assessed later may have been at different stages of natural symptom resolution at study entry. The interval between resolution of the acute infection and the baseline assessment was therefore recorded for each participant and is reported as median and interquartile range. Because the Friedman test does not accommodate covariates, the influence of this interval was examined in a sensitivity analysis correlating it (Spearman’s rho) with the magnitude of change between baseline and 12 months in the domains showing significant longitudinal change.

**Figure 1:**
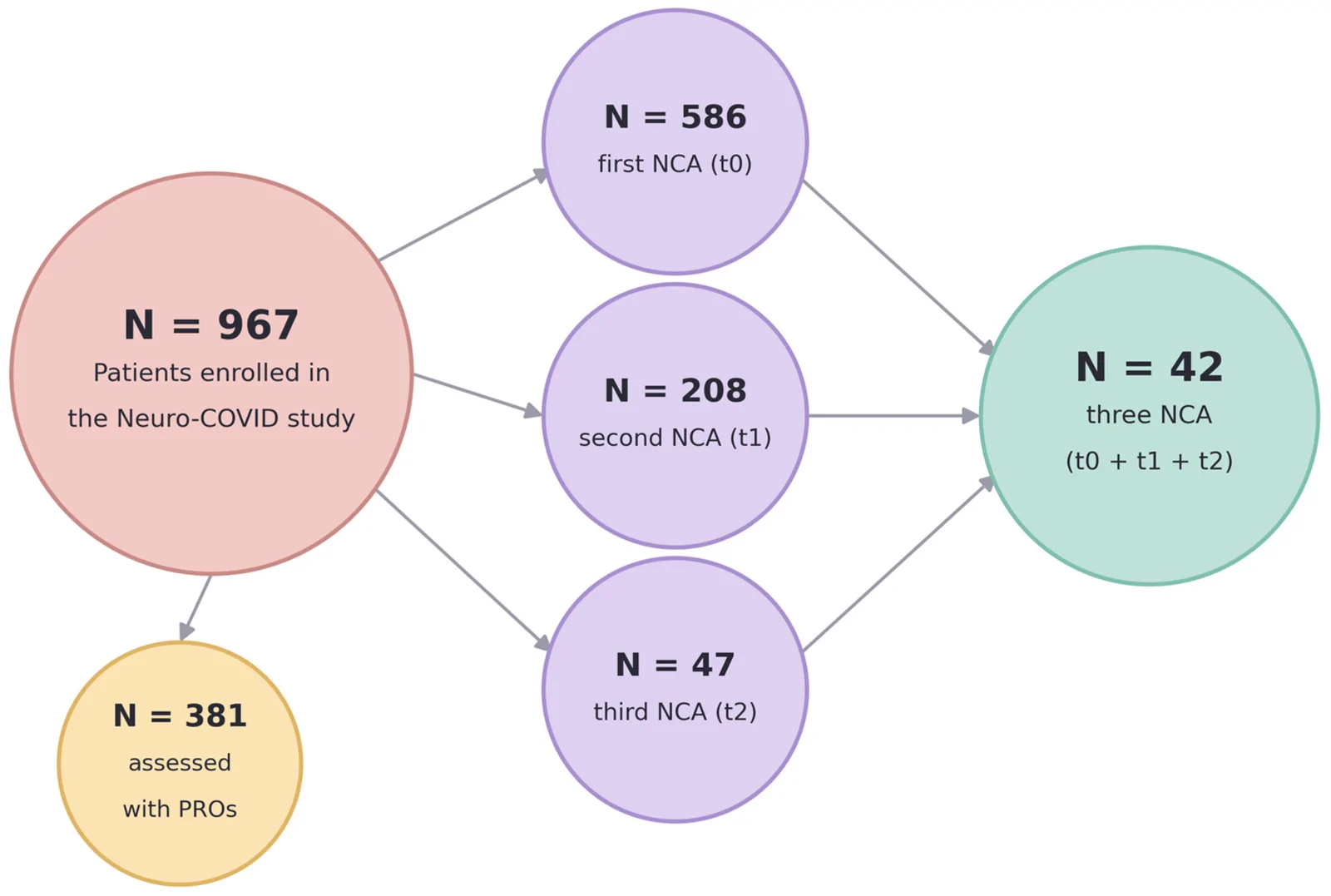
Flow chart of the study population. NCA = neurocognitive assessment; PROs = patient-reported outcomes; N = number of patients; t0, t1, t2 = assessments at approximately three, six and twelve months after resolution of the acute infection.

A flow diagram illustrating the study population and patient selection process is shown in Figure 1.

### 2.3. Neurocognitive Assessment

Neurocognitive assessment (NCA) was performed at three time points during follow-up: approximately three months (t0), six months (t1) and twelve months (t2) after the resolution of the acute infection.

Participants underwent a comprehensive standardized neuropsychological battery including 20 tests covering five cognitive domains: memory, attention, language, executive functions and psychomotor processing speed.

The battery was assembled to cover the five cognitive domains most consistently reported as affected after SARS-CoV-2 infection [24,25,26,27,28,29], selecting tests with Italian normative data allowing conversion into equivalent scores and, where possible, tests available in alternate forms permitting repeated administration. Global cognitive screening (MMSE) and a functional measure (IADL) were included to contextualise domain-specific performance. The following tests were administered:

Mini-Mental State Examination (MMSE) for global cognitive screening [30].

Rey Auditory Verbal Learning Test (RAVLT-ST, RAVLT-DR, RAVLT-REC) for verbal memory [31].

Digit Span Forward and Backward (DSF, DSB) for verbal short-term and working memory [32].

Corsi Span Forward and Backward (CSF, CSB) for visuospatial memory [32].

Rey-Osterrieth Complex Figure (ROCF-Copy and ROCF-Delayed Recall) for visuospatial constructional abilities and visual memory [33].

Trail Making Test A and B (TMTA, TMTB) for processing speed and executive functioning [34].

WAIS-R Digit Symbol (DS) for psychomotor processing speed [35].

Stroop Test (ST) for inhibitory control and cognitive flexibility [36].

Multiple Features Target Cancellation (MFTC) for attention [37].

Phonological and categorical verbal fluency tests (PVF, CVF) for language production [38].

Functional autonomy was evaluated using the Instrumental Activities of Daily Living scale (IADL) [39].

Raw test scores were adjusted for age, sex and educational level and converted into equivalent scores according to Italian normative standards [40,41]. Equivalent scores range from 0 to 4, where 0 denotes performance below the outer tolerance limit of the normative distribution and 4 performance within the upper normal range. Performance at each assessment was dichotomised as impaired (equivalent score = 0) or preserved (equivalent score ≥ 1), that is at or above the outer tolerance limit of the Italian normative distribution; the longitudinal analyses reported below were performed on this dichotomised classification. Only patients with complete data at all three assessments were analysed: no imputation was performed and no participant contributed partial data, so every analysis is based on the same 42 individuals at each time point.

### 2.4. Neuropsychiatric Assessment

The following questionnaires were administered to assess the presence of neuropsychiatric symptoms and their possible influence on neurocognitive performance: the Beck Anxiety Inventory (BAI) [42], which assesses the cognitive and physiological symptoms of anxiety [range = 0–63 were classified as in: 0–7 = no symptoms, 8–15 = mild symptoms, 16–25 = moderate symptoms; 26–63 = severe symptoms]; the Beck Depression Inventory (BDI-II) [43], which assesses cognitive, affective, and physiological symptoms of depression (Somatic-affective SA/Cognitive C dimensions) [range = 0–63 were classified as in: 0–13 = no symptoms, 14–19 = mild symptoms, 20–28 = moderate symptoms; 29–63 = severe symptoms]; the Pittsburgh Sleep Quality Index (PSQI) [44] for sleep quality assessment (if score > 5 indicates the presence of poor sleep quality). It should also be noted that several BDI-II items (e.g., fatigue, loss of energy, changes in sleep, appetite or weight, difficulty concentrating and reduced interest in activities) overlap substantially with the somatic and cognitive manifestations of PACS itself. Consequently, BDI-II scores in this population may partly capture the somatic burden of long COVID rather than depression per se, and depression severity may be overestimated. The use of the separate somatic-affective (SA) and cognitive (C) dimensions was intended to partially mitigate this issue.

### 2.5. Statistical Analysis

Data were summarized using descriptive statistics. Continuous variables are presented as medians and interquartile ranges (IQR), while categorical variables are expressed as frequencies and percentages.

Comparisons between categorical variables were performed using the Chi-square test or Fisher’s exact test when appropriate. Longitudinal changes in cognitive and psychiatric variables across the three time points were analysed using the non-parametric Friedman test.

Statistical significance was defined as *p* < 0.05. To account for the multiple comparisons inherent in the cognitive–neuropsychiatric association analyses, *p*-values were adjusted using the Benjamini–Hochberg false discovery rate (FDR) procedure, with a significance threshold set at FDR < 0.05. The same Benjamini–Hochberg procedure was subsequently applied across the twenty cognitive outcomes of the longitudinal analysis; both unadjusted and FDR-adjusted *p*-values are reported, the former being regarded as exploratory. Where a longitudinal change reached significance, pairwise post hoc comparisons between assessments were performed with McNemar’s test, Bonferroni-corrected for the three contrasts. Because the longitudinal variables were dichotomous, the Friedman test is algebraically equivalent to Cochran’s Q; results are reported as Friedman statistics for consistency with the software output. Effect sizes for longitudinal changes were quantified using Kendall’s W (interpreted as small ≥ 0.10, medium ≥ 0.30, large ≥ 0.50). All analyses were conducted using SPSS version 29.0 (IBM Corp., Chicago, IL, USA).

To minimize practice (test–retest) effects arising from repeated assessment, alternate (parallel) forms were administered across the three time points for those tests for which they exist: the Rey Auditory Verbal Learning Test, the copy of the Rey-Osterrieth Complex Figure, and Trail Making Test A and B. For the remaining tests the same version was readministered. The use of alternate forms reduces, but does not eliminate, the influence of task-specific learning on serial performance: procedural familiarity with the testing situation itself may still contribute to improved scores, and this should be borne in mind when interpreting the observed changes.

## 3. Results

### Participant Characteristics

A total of 42 patients were included in the analysis. The median age was 57 years (IQR 38–81); 15 participants (35.7%) were female and 27 (64.3%) male. The median educational level was 13 years (IQR 8–18). Most patients had been previously hospitalized during the acute phase of COVID-19 (81%), and 66.7% required oxygen therapy. Of the 967 patients initially enrolled at the Post-COVID outpatient service, only 42 (4.3%) completed all three neurocognitive assessments and were included in the present longitudinal analysis (see Figure 1). Reasons for non-completion were established retrospectively from the clinical records of the outpatient service and from the impressions of the clinicians managing follow-up; they were not collected systematically through structured contact with patients who discontinued, and should therefore be regarded as indicative rather than quantified. They included perceived cognitive recovery leading to voluntary dropout, lack of motivation to return in the absence of ongoing symptoms, severe psychological distress precluding continued participation, and work or logistical constraints limiting availability for repeated testing.

To characterise potential selection bias, baseline demographic and clinical characteristics of study completers were formally compared with those of non-completers (n = 544; Table S1). Age (completers: median 57 years, IQR 51–60; non-completers: median 54 years, IQR 47–61; *p* = 0.273) and educational level (both groups: median 13 years; *p* = 0.359) did not differ significantly between groups. Notably, however, completers had a significantly higher prevalence of acute-phase respiratory support (66.7% vs. 33.6%; *p* < 0.001), pulmonary embolism (16.7% vs. 2.9%; *p* < 0.001), corticosteroid use (66.7% vs. 47.4%; *p* = 0.016), and tocilizumab/sarilumab treatment (7.1% vs. 1.1%; *p* = 0.021), and were more frequently male (64.3% vs. 44.7%; *p* = 0.014). These findings indicate that completers were, if anything, more severely ill during the acute phase than non-completers, rather than representing a healthier or more motivated subgroup. The direction of any resulting selection bias is therefore likely to be towards overrepresentation of severe acute disease and its sequelae, which should be considered when interpreting the observed cognitive trajectories. This interpretation assumes that documented acute severity reflects true severity, which it may not. Patients with overt complications such as pulmonary embolism were identified and treated, whereas undiagnosed distal microvascular involvement would leave a patient classified as less severe while potentially contributing to persistent impairment [9,10,11,12]. Recorded severity should therefore be read as a proxy for the intensity of acute care received rather than as a direct measure of biological insult.

Baseline demographic and clinical characteristics of the study population are summarized in Table 1.

Longitudinal neurocognitive outcomes.

Longitudinal analyses revealed significant changes in several cognitive domains across the three assessments.

Improvements in verbal short-term learning (RAVLT-ST) were observed over time, with the proportion of impaired patients decreasing from 23.8% at baseline (t0) to 11.9% at t1 and 9.5% at t2 (*p* = 0.032).

Similarly, visuospatial short-term memory (CSF) improved significantly, with impairment rates decreasing from 31% at t0 to 2.4% at t1 and 0% at t2 (*p* < 0.0001). Improvements were also observed in visuospatial working memory (CSB) (*p* = 0.002).

Significant improvements were additionally observed in constructional praxis (ROCF-C; *p* = 0.018), phonological verbal fluency (PVF; *p* = 0.05) and psychomotor processing speed (DS; *p* = 0.002).

Sleep quality also significantly improved across follow-up evaluations, with the proportion of patients reporting poor sleep quality decreasing from 31% at baseline to 7.1% at t1 and 2.4% at t2 (*p* < 0.0001).

Detailed results of the longitudinal neurocognitive and neuropsychiatric analyses are presented in Table 2. Six of the twenty cognitive outcomes changed significantly before correction for multiple comparisons. After Benjamini–Hochberg adjustment across the twenty outcomes, three remained significant: visuospatial short-term memory (CSF; FDR-adjusted *p* = 0.001), visuospatial working memory (CSB; *p* = 0.013) and psychomotor processing speed (DS; *p* = 0.013); the changes in verbal short-term learning (RAVLT-ST; *p* = 0.128), constructional praxis (ROCF-C; *p* = 0.090) and phonological verbal fluency (PVF; *p* = 0.167) did not survive adjustment and are reported as exploratory. Post hoc pairwise comparisons for the three robust outcomes located the change entirely in the first interval: all three improved significantly between baseline and 12 months (CSF *p* = 0.001; CSB *p* = 0.022; DS *p* = 0.010), CSF and DS improved significantly between baseline and 6 months (*p* = 0.002 and *p* = 0.038), and none changed between 6 and 12 months (all *p* = 1.000). The interval between resolution of the acute infection and the baseline assessment had a median of 2.9 months (IQR 2.8–5.6, range 1.3–7.6). This interval was not significantly associated with the magnitude of improvement between baseline and 12 months in any of the three robust outcomes (CSF rho = −0.28, *p* = 0.124; CSB rho = −0.21, *p* = 0.259; DS rho = −0.32, *p* = 0.074), indicating that heterogeneity in the timing of the first evaluation does not account for the observed change.

Association between neurocognitive performance and neuropsychiatric symptoms.

The following analyses of associations between neurocognitive performance and neuropsychiatric symptoms are considered exploratory, given the multiple comparisons and small sample size. *p*-values were adjusted using the Benjamini–Hochberg FDR procedure; all reported associations survived FDR correction (FDR < 0.05).

At baseline (t0), increased anxiety symptoms were significantly associated with poorer performance in verbal short-term memory (DSF; *p* = 0.040) and psychomotor processing speed (DS; *p* = 0.020). Sleep disturbances were also associated with reduced processing speed (*p* = 0.018).

At the second assessment (t1), anxiety symptoms were associated with poorer verbal learning performance (RAVLT-ST; *p* = 0.035), while sleep disturbances were associated with poorer performance in both verbal learning (RAVLT-ST; *p* = 0.033) and verbal long-term memory (RAVLT-DR; *p* = 0.010).

At the final assessment (t2), depressive symptoms were associated with poorer performance in verbal long-term memory (RAVLT-DR).

**Figure 2:**
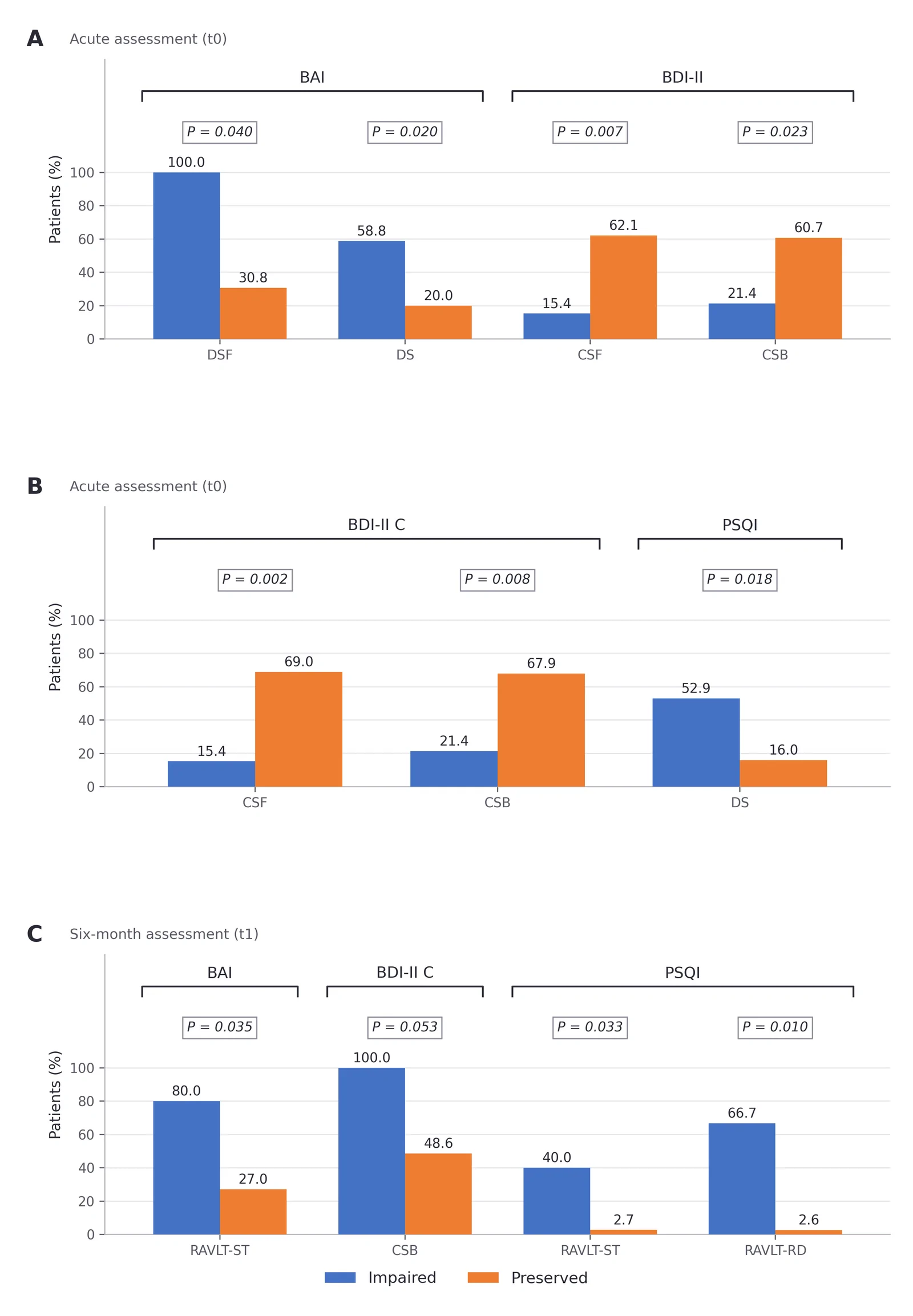
Association between neurocognitive performance and neuropsychiatric symptoms. (**A**) Acute assessment (T0), Beck Anxiety Inventory (BAI) and Beck Depression Inventory-II (BDI-II). (**B**) Acute assessment (T0), BDI-II cognitive subscale (BDI-II C) and Pittsburgh Sleep Quality Index (PSQI). (**C**) Six-month assessment (T1). Bars show the percentage of patients classified as impaired (equivalent score = 0) and preserved on each test, within the group scoring above the clinical threshold on the corresponding questionnaire. P values are from [test]. CSB, Corsi span backward; CSF, Corsi span forward; DS, Digit Symbol; DSF, Digit span forward; RAVLT-RD, Rey Auditory Verbal Learning Test delayed recall; RAVLT-ST, Rey Auditory Verbal Learning Test short-term.

A detailed representation of these associations is provided in Figure 2A–C.

## 4. Discussion

To our knowledge, this study represents one of the few longitudinal investigations providing detailed neuropsychological monitoring across multiple cognitive domains in patients with PACS using in-person standardized assessment.

This longitudinal study investigated the evolution of neurocognitive performance and neuropsychiatric symptoms in patients with PACS undergoing comprehensive neuropsychological assessment over a one-year follow-up period. Our findings indicate that cognitive performance may gradually improve over time in several domains, particularly verbal short-term learning, visuospatial memory, working memory, constructional praxis, phonological verbal fluency and psychomotor processing speed. However, recovery was heterogeneous across cognitive domains, and a subset of individuals continued to show impaired or borderline performance during follow-up.

These findings are consistent with previous studies reporting partial recovery of cognitive function after COVID-19, in which deficits persisting for months tend to improve progressively in a proportion of patients [45,46,47,48], particularly in attentional and executive functions, while residual impairments remain in some individuals [13,20]. These improvements should nonetheless be interpreted with caution. The use of alternate (parallel) test forms across assessments makes test–retest learning an unlikely explanation, supporting a genuine change in performance. However, most participants had been hospitalized, frequently with oxygen and steroid treatment. Substantial cognitive improvement during the year after hospital discharge is well documented across critically ill populations independently of SARS-CoV-2. As detailed in the Limitations, the attrition pattern in our cohort (Table S1) selectively overrepresents patients whose acute illness was more severe as documented in the clinical record, which is not necessarily the same as biologically more severe disease. The early entry criterion (from four weeks after infection) further increases the likelihood that part of the improvement reflects natural resolution of acute illness rather than a chronic PACS-specific process. Our design cannot separate these two contributions. Recent large longitudinal studies are consistent with this pattern: in a prospective in-person cohort, most cognitive domains improved progressively while processing speed and executive functioning remained below the normative mean [49], and serial assessments in the COVID and Cognition Study similarly described gradual symptom and cognitive change over time [24].

The mechanisms underlying cognitive dysfunction in PACS are likely multifactorial. Neuroinflammatory responses triggered by SARS-CoV-2 have been proposed as a major contributor to persistent neurological symptoms [20], potentially affecting neuronal function, synaptic transmission and cerebral microcirculation, while systemic factors such as hypoxia, endothelial dysfunction and metabolic disturbances during acute infection may also contribute to neural injury [14,15,16,17]. However, as the present study did not include neuroimaging or inflammatory biomarkers, these pathways were not measured and no causal link can be established. The gradual improvement seen in our cohort is compatible with, but cannot be attributed to, the progressive resolution of inflammatory processes; alternative explanations, including expected post-hospitalization recovery and practice effects, are at least equally plausible.

Two domains showed no improvement at all. Categorical fluency was not only stable but showed a higher proportion of impaired patients at 12 months than at 6 months, which argues against low test sensitivity and points instead to a genuinely persistent deficit in lexical-semantic retrieval. Attentional accuracy on the MFTC showed a ceiling pattern, with most patients performing within normal limits at every assessment, leaving little room for detectable improvement. Improvements were not uniform across domains: while several functions recovered, others remained stable. Improvement was moreover not uniform in direction: in one domain—verbal short-term span—the proportion of impaired patients increased across assessments, from 7.1% at t0 to 11.9% at t1 and 16.7% at t2, and category fluency also worsened between the second and third evaluation. Similar heterogeneity has been reported previously, with cognitive trajectories varying substantially between individuals [13,25]. A recent 36-month study of hospitalized COVID-19 patients identified four distinct trajectories—stable normal function, recovery, persistent impairment and delayed decline [25]—while other long-term data reported essentially negligible change in most patients [26], confirming that the magnitude and direction of cognitive change remain heterogeneous across studies. These patterns may reflect differences in disease severity, individual vulnerability, pre-existing conditions or psychological factors.

Sleep quality improved significantly over time. Sleep disturbances are among the most prevalent symptoms in PACS [27,28] and may contribute to cognitive complaints by affecting attention, memory consolidation and executive functioning; the observed improvement may therefore have played a role in the recovery of certain cognitive functions, as sleep disturbances and mental fatigue—the latter understood here as the subjective difficulty of sustaining cognitive effort over time, a construct drawn from the literature and not measured with a dedicated instrument in the present study—are closely related to performance in tasks involving processing speed and attentional resources [22,29].

Our results also highlight the complex relationship between neurocognitive performance and neuropsychiatric symptoms in patients with PACS. Anxiety symptoms were associated with poorer performance in verbal short-term memory and psychomotor processing speed during the first assessment. In addition, sleep disturbances were associated with reduced performance in processing speed and verbal memory tasks during follow-up. These findings are consistent with previous research indicating that psychological distress may contribute to cognitive impairment in patients following COVID-19 [25,50,51]. The direction of this relationship cannot be established from our data. Several items of the BAI capture autonomic symptoms—palpitations, breathlessness, dizziness, feelings of instability—that are also cardinal features of the dysautonomia frequently described in PACS. Anxiety scores in this population may therefore partly index autonomic dysfunction rather than anxiety per se, in the same way that BDI-II scores may index somatic burden.

The relationship between depressive symptoms and cognition changed across assessments: at baseline, depressive symptoms were unexpectedly associated with better visuospatial memory (CSF/CSB), whereas later evaluations showed an association with poorer verbal long-term memory. The counter-intuitive baseline association is most likely spurious, given the small sample, the many cognitive–neuropsychiatric pairs tested without correction for multiple comparisons, and dichotomized categories with few impaired cases. All associations were tested using Chi-square or Fisher’s exact test (expected counts < 5) on dichotomised variables, with n = 42 at each time point, and should be regarded as hypothesis-generating only. These fluctuations may reflect the bidirectional interaction between mood and cognition, whereby psychological distress influences cognition through reduced motivation, impaired attentional control or mental fatigue, while persistent cognitive difficulties may maintain anxiety and depressive symptoms.

From a clinical perspective, these findings emphasize the importance of comprehensive neuropsychological assessment in PACS, as cognitive complaints may reflect both objective alterations and psychological factors. Long-term monitoring may help identify individuals at risk of persistent impairment. Where impairment persists, cognitive rehabilitation targeting the specific domains involved may be considered, although its efficacy in PACS has not been established. Rehabilitation alone is unlikely to be sufficient where symptoms are sustained by neuroinflammatory or neurovascular mechanisms, and pharmacological treatment of comorbid depression, anxiety and sleep disturbance, as well as management of autonomic symptoms, should be considered as part of an integrated approach.

All participants for whom the date of acute infection was documented were infected between March 2020 and January 2021, which is before the emergence of the Delta and Omicron lineages and before vaccination was available. No patient in the cohort was infected during the Delta or Omicron periods, so stratification by dominant variant was neither possible nor informative; the extended enrolment window reflects the timing of referral to the Post-COVID service rather than heterogeneity of the infecting variant. Several limitations should be considered. First, the study did not include pre-infection neurocognitive assessments, limiting estimation of cognitive decline attributable to SARS-CoV-2. Second, the absence of a matched control group prevents comparison with individuals without prior COVID-19. Concomitant treatments received during follow-up—cognitive rehabilitation, psychological support, antidepressant or hypnotic medication—were not systematically recorded, so the extent to which the observed improvement reflects unassisted change rather than the effect of concurrent care cannot be determined. Because completers were more severely affected during the acute phase than those who discontinued, their baseline scores were on average more extreme, and regression to the mean is a plausible partial contributor to the improvement observed; our design cannot separate it from genuine recovery. Third, the small sample size limits statistical power and generalizability: only 42 of 967 enrolled patients (4.3%) completed all three assessments. As shown in Table S1, completers did not differ from non-completers in age or education but were more severely ill acutely (respiratory support 66.7% vs. 33.6%, *p* < 0.001; pulmonary embolism 16.7% vs. 2.9%, *p* < 0.001; corticosteroids 66.7% vs. 47.4%, *p* = 0.016), so the sample is enriched for hypoxia-related and post-critical-illness trajectories rather than healthier individuals. Fourth, the cohort was older than the broader population of patients attending the Post-COVID service, from which our completers represent a small and selected subgroup, and was predominantly hospitalized, two-thirds receiving oxygen and steroids; cognitive dysfunction and its improvement may be driven largely by hypoxia-related injury, critical illness and expected post-hospitalization recovery common across non-COVID populations, rather than PACS-specific mechanisms, while the high prevalence of hypertension (42.9%) and vascular risk factors may have contributed through a vascular pathway. These features limit generalizability to the broader, mostly non-hospitalized long-COVID population with brain fog. Fifth, no neuroimaging or inflammatory biomarker data were available, so mechanistic interpretations remain speculative. Finally, the inclusion criterion of symptoms from four weeks after infection reflects an earlier, broader definition of PACS than the three-month criterion now recommended by the WHO, NASEM and CDC, and the variable baseline window (up to seven months) adds heterogeneity in the timing of first evaluation. Finally, the cohort was infected in 2020, before vaccination was available and before the emergence of the Omicron and subsequent variants. Both vaccination prior to infection and infection with more recent variants have been associated with a lower burden of post-acute symptoms, and our findings should not be generalised to patients infected under those conditions.

Despite these limitations, the present study provides valuable longitudinal data based on detailed in-person neuropsychological assessments across multiple cognitive domains. The use of a comprehensive neuropsychological battery and repeated evaluations allowed a more nuanced characterization of cognitive trajectories in patients with PACS.

Future studies should aim to include larger multicentre cohorts and appropriate control groups in order to better clarify the mechanisms and long-term trajectory of cognitive impairment following COVID-19. In addition, further research integrating neuroimaging, inflammatory biomarkers and neuropsychological assessment may help elucidate the biological processes underlying cognitive dysfunction in PACS.

## 5. Conclusions

Our results provide descriptive longitudinal evidence suggesting that, in a small and predominantly hospitalized cohort with post-acute COVID-19 syndrome, performance on several cognitive measures tended to improve over the first year of follow-up, while deficits in a subset of cognitive functions persisted. Critically, a formal comparison showed that study completers were more severely ill during the acute phase than non-completers, indicating that the sample selectively represents patients with more severe acute disease rather than those who recovered most easily; the observed improvements may therefore partly reflect the expected post-hospitalisation recovery trajectory rather than PACS-specific processes. The use of alternate test forms across assessments makes a substantial contribution of practice effects unlikely. Exploratory analyses indicated that anxiety, depressive symptoms and sleep disturbances were associated with poorer performance across several cognitive domains; these findings should nonetheless be regarded as hypothesis-generating only. Integrated neuropsychological and psychiatric monitoring appears warranted in this population. Larger controlled studies with non-hospitalised comparison groups are needed to clarify the specific contribution of PACS to the observed cognitive trajectories.

## Figures and Tables

**Table 1:** Baseline clinical characteristics for the study population.

Variables	Sample N = 42
Age, median (IQR)	57 (38–81)
Female	15 (35.7%)
Education, median (IQR)	13 (8–18)
Smoking	2 (4.8%)
Comorbidities	
Previous acute myocardial infarction	2 (4.8%)
Hypertension	18 (42.9%)
Diabetes	5 (11.9%)
Cardiac Disease	3 (7.1%)
Neurological disease	2 (4.8%)
Respiratory diseases	0 (0%)
Cancer	0 (0%)
Neuropsychological symptoms	31 (73.8%)
Oxygen therapy	28 (66.7%)
Pulmonary Embolism	7 (16.7%)
Previous hospitalization (PH)	34 (81.0%)
Anti-SARS-CoV-2 therapy	
Tocilizumab/sarilumab	3 (7.1%)
Kaletra	4 (9.5%)
Steroids	28 (66.7%)
Remdesivir	20 (47.6%)
Plasma	1 (2.4%)
Hydroxychloroquine	9 (21.4%)
LMWH	28 (66.7%)
ACE_I	6 (14.3%)
ARB	2 (4.8%)
Statine	5 (11.9%)

**Table 2:** Temporal changes in cognitive functions and neuropsychiatric symptoms (T0-T1-T2).

	T0		T1		T2		*p* Value
NCA	normal	impaired	normal	impaired	normal	impaired	
MMSE	42 (100%)	0	42 (100%)	0	42 (100%)	0	1
RAVLT-ST	32 (76.2%)	10 (23.8%)	37 (88.1%)	5 (11.9%)	38 (90.5%)	4 (9.5%)	**0.032**
RAVLT-DR	36 (85.7%)	6 (14.3%)	39 (92.9%)	3 (7.1%)	39 (92.9%)	3 (7.1%)	0.276
RAVLT-REC	30 (71.4%)	12 (28.6%)	31 (73.8%)	11 (26.2%)	34 (81%)	8 (19%)	0.486
ROCF-DR	27 (64.3%)	15 (35.7%)	31 (73.8%)	11 (26.2%)	32 (76.2%)	10 (23.8%)	0.148
DSF	39 (92.9%)	3 (7.1%)	37 (88.1%)	5 (11.9%)	35 (83.3%)	7 (16.7%)	0.223
DSB	38 (90.5%)	4 (9.5%)	36 (85.7%)	6 (14.3%)	40 (95.2%)	2 (4.8%)	0.264
CSF	29 (69%)	13 (31%)	41 (97.6%)	1 (2.4%)	42 (100%)	0	**<0.0001**
CSB	28 (66.7%)	14 (33.3%)	37 (88.1%)	5 (11.9%)	39 (92.9%)	3 (7.1%)	**0.002**
ROCF-C	35 (83.3%)	7 (16.7%)	39 (92.9%)	3 (7.1%)	41 (97.6%)	1 (2.4%)	**0.018**
MFTC-ACC	30 (71.4%)	12 (28.6%)	36 (85.7%)	6 (14.3%)	36 (85.7%)	6 (14.3%)	0.105
MFTC-ERR	42 (100%)	0	42 (100%)	0	42 (100%)	0	1
MFTC-T	42 (100%)	0	42 (100%)	0	42 (100%)	0	1
PVF	34 (81%)	8 (19%)	36 (85.7%)	6 (14.3%)	38 (90.5%)	4 (9.5%)	**0.05**
CVF	26 (61.9%)	16 (38.1%)	32 (76.2%)	10 (23.8%)	30 (71.4%)	12 (28.6%)	0.116
ST-T	41 (97.6%)	1 (2.4%)	39 (92.9%)	3 (7.1%)	41 (97.6%)	1 (2.4%)	0.368
ST-ERR	40 (95.2%)	2 (4.8%)	42 (100%)	0	42 (100%)	0	0.135
TMTA	41 (97.6%)	1 (2.4%)	42 (100%)	0	42 (100%)	0	0.368
TMTB	42 (100%)	0	42 (100%)	0	42 (100%)	0	1
DS	25 (59.5%)	17 (40.5%)	36 (85.7%)	6 (14.3%)	36 (85.7%)	6 (14.3%)	**0.002**
BAI	27 (64.3%)	15 (35.7%)	28 (66.7%)	14 (33.3%)	30 (71.4%)	12 (28.6%)	0.705
BDI-II	22 (52.4%)	20 (47.6%)	26 (61.9%)	16 (38.1%)	25 (59.5%)	17 (40.5%)	0.395
BDI-II SA	24 (57.1%)	18 (42.9%)	29 (69%)	13 (31%)	29 (69%)	13 (31%)	0.249
BDI-II C	20 (47.6%)	22 (52.4%)	19 (45.2%)	23 (54.8%)	23 (54.8%)	19 (45.2%)	0.504
PSQI	29 (69%)	13 (31%)	39 (92.9%)	3 (7.1%)	41 (97.6%)	1 (2.4%)	**<0.0001**

Note. For each test, the number and percentage of patients classified as impaired (equivalent score = 0) at each assessment are reported; these proportions are descriptive. *p* values are derived from the Friedman test computed across the three time points on the dichotomised impaired/preserved classification displayed here. Abbreviations: Mini Mentale State Examination (MMSE); Rey Auditory Verbal Learning Test-Short Term (RAVLT-ST); Rey Auditory Verbal Learning Test-Delayed Recall (RAVLT-RD); Rey Auditory Verbal Learning Test-Recognition (RAVLT-REC); Rey-Osterrieth Complex Figure- Delayed Recall (ROCF-DR); Digit Span Forward (DSF); Digit Span Backward (DSB); Corsi Span Forward (CSF); Corsi Span Backward (CSB); Rey-Osterrieth Complex Figure- Copy (ROCF-C); Multiple Features Target Cancellation-Accuracy (MFTC-ACC); Multiple Features Target Cancellation-Errors (MFTC-ERR); Multiple Features Target Cancellation-Time (MFTC-T); Phonological Verbal Fluency (PVF); Categorical Verbal Fluency (CVF); Stroop test Color Word- Time (ST-T); Stroop test Color Word- Errors (ST-ERR); Trial Making Test A (TMTA); Trial Making Test B (TMTB);Digit Symbol Test (DS); Beck Anxiety Inventory (BAI); Beck Depression Inventory (BDI-II); Beck Depression Inventory Somatic-Affective (BDI-II SA); Beck Depression Inventory Cognitive (BDI-II C); Pittsburgh Sleep Quality Index (PSQI).

## Data Availability

The datasets generated and/or analyzed during the current study are not publicly available due to patient privacy reasons, but are available from the corresponding author on reasonable request.

## Supplementary Materials

The following supporting information can be downloaded at https://www.mdpi.com/article/10.3390/neurolint18090163/s1, Table S1. Baseline characteristics of study completers (n = 42) versus non-completers (n = 544). Completers were patients who attended all three neurocognitive assessments; non-completers attended the first evaluation only or did not complete the twelve-month follow-up. Continuous variables are reported as median (interquartile range) and compared with the Mann–Whitney U test; categorical variables are reported as n (%) and compared with the chi-square or Fisher exact test as appropriate. Values in bold indicate *p* < 0.05. All comorbidities and chronic medications were documented prior to SARS-CoV-2 infection; acute-phase characteristics and treatments refer to the index COVID-19 episode. ACE, angiotensin-converting enzyme; ARB, angiotensin II receptor blocker; CPAP, continuous positive airway pressure; IQR, interquartile range; NIV, non-invasive ventilation; O2, oxygen therapy.

## Abbreviations

The following abbreviations are used in this manuscript:

BAI	Beck Anxiety Inventory LMWH: Low Molecular Weight Heparin
BDI-II	Beck Depression Inventory-II
C	Cognitive (BDI-II subscale)
CDC	Centers for Disease Control and Prevention
CSB	Corsi Span Backward
CSF	Corsi Span Forward
CVF	Categorical Verbal Fluency
DS	Digit Symbol (WAIS-R)
DSB	Digit Span Backward
DSF	Digit Span Forward
FDR	False Discovery Rate
IADL	Instrumental Activities of Daily Living
LC	Long COVID
IQR	Interquartile Range
MFTC	Multiple Features Target Cancellation
MFTC-ACC	Multiple Features Target Cancellation—Accuracy
MFTC-ERR	Multiple Features Target Cancellation—Errors
MFTC-T	Multiple Features Target Cancellation—Time
MMSE	Mini-Mental State Examination
NASEM	National Academies of Sciences, Engineering, and Medicine
NCA	Neurocognitive Assessment
NICE	National Institute for Health and Care Excellence
PCC	Post COVID-19 Condition
PACS	Post-Acute COVID-19 Syndrome
PSQI	Pittsburgh Sleep Quality Index
PVF	Phonological Verbal Fluency
RAVLT	Rey Auditory Verbal Learning Test
RAVLT-DR	Rey Auditory Verbal Learning Test—Delayed Recall
RAVLT-REC	Rey Auditory Verbal Learning Test—Recognition
RAVLT-ST	Rey Auditory Verbal Learning Test—Short-Term learning
ROCF	Rey-Osterrieth Complex Figure
ROCF-C	Rey-Osterrieth Complex Figure—Copy
ROCF-DR	Rey-Osterrieth Complex Figure—Delayed Recall
SA	Somatic-Affective (BDI-II subscale)
SARS-CoV-2	Severe Acute Respiratory Syndrome Coronavirus 2
ST	Stroop Test
ST-ERR	Stroop Test—Errors
ST-T	Stroop Test—Time
TMTA	Trail Making Test, Part A
TMTB	Trail Making Test, Part B
WAIS-R	Wechsler Adult Intelligence Scale—Revised
WHO	World Health Organization
