# Supplementary material for: Neurocognitive and Neuropsychiatric Trajectories in a Post-COVID Cohort: A Descriptive Longitudinal Study
Source: Neurol Int. 2026 Aug 24;18(9):163. doi: 10.3390/neurolint18090163 (PMC13610980; doi:10.3390/neurolint18090163)
Supplement: Supplementary file 1 [file neurolint-18-00163-s001.zip › neurolint-4487471-supplementary.pdf]

**Supplementary Table S1.** Baseline characteristics of study completers (n=42) versus non-completers (n=544).

| Characteristic | Completers\n(n=42) | Non-completers\n(n=544) | p-value |
|---|---|---|---|
| **Demographics** | | | |
| Ag e (years), median (IQR) | 57 (51–60) | 54 (47–61) | 0.273 |
| Education (years), median (IQR) | 13 (13–18) | 13 (13–18) | 0.359 |
| **Comorbidities** | | | |
| Female sex, n (%) | **27 (64.3%)** | **243 (44.7%)** | **0.014** |
| Smoker, n (%) | 2 (4.8%) | 24 (4.4%) | 0.709 |
| Prior myocardial infarction, n (%) | 2 (4.8%) | 4 (0.7%) | 0.063 |
| Heart failure history, n (%) | 3 (7.1%) | 14 (2.6%) | 0.115 |
| Hypertension, n (%) | 18 (42.9%) | 159 (29.2%) | 0.064 |
| Diabetes, n (%) | 5 (11.9%) | 43 (7.9%) | 0.362 |
| Neurological comorbidities, n (%) | 2 (4.8%) | 10 (1.8%) | 0.210 |
| Neurological symptoms during acute phase, n (%) | 31 (73.8%) | 384 (70.6%) | 0.658 |
| **Acute phase characteristics & treatment** | | | |
| Any respiratory support (NIV/CPAP/O2), n (%) | **28 (66.7%)** | **183 (33.6%)** | **<0.001** |
| Pulmonary embolism, n (%) | **7 (16.7%)** | **16 (2.9%)** | **<0.001** |
| Corticosteroids, n (%) | **28 (66.7%)** | **258 (47.4%)** | **0.016** |
| Tocilizumab/sarilumab, n (%) | **3 (7.1%)** | **6 (1.1%)** | **0.021** |
| Lopinavir/ritonavir, n (%) | 4 (9.5%) | 113 (20.8%) | 0.107 |
| Hydroxychloroquine, n (%) | 9 (21.4%) | 73 (13.4%) | 0.149 |
| **Chronic medications** | | | |
| ACE inhibitors, n (%) | 6 (14.3%) | 39 (7.2%) | 0.095 |
| ARB, n (%) | 2 (4.8%) | 47 (8.6%) | 0.565 |
| Statins, n (%) | 5 (11.9%) | 28 (5.1%) | 0.067 |
